# Micro-Scale Vacuum Compression Molding as a Predictive Screening Tool of Protein Integrity for Potential Hot-Melt Extrusion Processes

**DOI:** 10.3390/pharmaceutics15030723

**Published:** 2023-02-22

**Authors:** Katharina Dauer, Karl G. Wagner

**Affiliations:** Department of Pharmaceutics, Pharmaceutical Institute, University of Bonn, 53121 Bonn, Germany

**Keywords:** vacuum compression molding, screening tool, formulation development, protein stability, protein characterization, biopharmaceuticals, solid-state stability

## Abstract

Hot-melt extrusion (HME) is used for the production of solid protein formulations mainly for two reasons: increased protein stability in solid state and/or long-term release systems (e.g., protein-loaded implants). However, HME requires considerable amounts of material even at small-scale (>2 g batch size). In this study, we introduced vacuum compression molding (VCM) as a predictive screening tool of protein stability for potential HME processing. The focus was to identify appropriate polymeric matrices prior to extrusion and evaluation of protein stability after thermal stress using only a few milligrams of protein. The protein stability of lysozyme, BSA, and human insulin embedded in PEG 20,000, PLGA, or EVA by VCM was investigated by DSC, FT-IR, and SEC. The results from the protein-loaded discs provided important insights into the solid-state stabilizing mechanisms of protein candidates. We demonstrated the successful application of VCM for a set of proteins and polymers, showing, in particular, a high potential for EVA as a polymeric matrix for solid-state stabilization of proteins and the production of extended-release dosage forms. Stable protein-polymer mixtures with sufficient protein stability after VCM could be then introduced to a combination of thermal and shear stress by HME and further investigated with regard to their process-related protein stability.

## 1. Introduction

The global biopharmaceutical sector is growing, and is developing innovative medicines that address a wide range of therapies for severe diseases and other human-related needs [1,2]. Moreover, the formulation development process of many biologics often wastes time and resources, and the amount of a peptide or protein candidate in early development is limited (i.e., a few milligrams) [3]. These aspects also need to be considered when evaluating alternative formulations, formulation strategies, and modes of administration. In particular, solid biopharmaceutics for peroral, nasal, or parenteral administration would significantly expand or improve the therapeutic use and the compliance of protein-based drugs [4,5,6]. Furthermore, solid protein and peptide formulations offer the potential for increased stability and underline the importance of alternative solid protein formulations (e.g., protein-loaded extrudates or implants). In addition to protein stability issues, an early evaluation and predictive screening of composition and formulation process parameters on a micro-scale level is of crucial importance, as, due to the costs of proteins, conventional development programs are financially unfeasible. The aim is to enable a good and early starting point for the production of solid protein formulations with sufficient protein stability and prior cost-intensive protein formulation development of long-term release systems. The stabilization of proteins can be achieved by embedding the protein or peptide powder in eroding or dissolving polymer matrices by scalable formulation technologies such as hot-melt extrusion (HME), freeze-drying (FD), or spray-freeze drying (SFD) [7,8,9]. HME was introduced with increasing awareness in several works as a manufacturing tool for the production of solid protein formulations, for instance, as a solid-state stabilization approach or for the development of long-term release systems for parenteral administration (e.g., protein-loaded implants) [8,10,11,12]. The use of HME provides a variety of motives for the formulation of long-term delivery systems: (i) no use of organic solvents, (ii) high drug loadings in polymeric matrices are feasible, (iii) simple scale-up, and (iv) use of additional excipients such as surfactants are not mandatory [13,14]. Different types of polymers can be simply used to embed proteins or peptides in a matrix for solid-state stabilization and optimizing the protein release behavior by HME processing [8,10]. Furthermore, the used polymers can ensure the stable folded structure of a protein or peptide during the HME process due to an immobilization of active biomolecules in the polymeric matrix [15,16]. However, during HME, the protein and peptide molecules are exposed to a combination of temperature and shear stress (designated as thermomechanical stress). Both can affect or initiate an unfolding of the protein or its degradation, resulting in an irreversible aggregation accompanied by a potential loss of protein functionality [15]. In a previously described study, we presented a sensitive characterization pathway of protein stability focused on the characterization of protein-loaded extrudates at a high drug load (i.e., >40%) prepared by ram extrusion and twin-screw extrusion (TSE). Here, changes in protein stability affected by thermomechanical stress during the extrusion process were investigated [10]. Although small-scale HME leads to high yields of an extruded powder mixture, the process is still associated with a moderate material input (i.e., >2 g of the powdered protein-polymer mixture) and material loss (e.g., <0.5 g of the protein-polymer mixture due to gaps between the rotating screws in TSE processing). Additionally, innovative formulation and screening tools in micro-scale (mg-scale) are highly desirable especially in the early phase of formulation development. Furthermore, a fundamental formulation development strategy is based on an extensive knowledge of the physicochemical peptide and protein stability accompanied with appropriate analytical methods to evaluate the process-related stability of active biomolecules in different formulations. This study proposes the implementation of micro-scale vacuum compression molding (VCM) as a predictive screening tool of protein integrity and polymers by overcoming the major drawback of conventional HME screening by using a minimum quantity of API and material. VCM was introduced by Treffer et al. [17] and is a novel, fusion-based tool for the simple preparation of thermoplastic specimens starting from powders, where the molten samples are evacuated and compressed into a defined solid form (e.g., cylindrical discs or implants) without any air inclusions or voids [17]. Additionally, the design of the VCM tool reduces the preparation time of the samples compared to HME processing, which requires time for assembling of extruder components, adjusting constant feeding rates, and cleaning [17]. Drug-loaded implants are one of the promising solid dosage forms for parenteral extended release [15,18]. However, the design of such implants for sustained release of proteins is a complex challenge, achieving protein solid-state stabilization in combination with a micro-scale screening tool, which is predictive for further HME processing. We used three polymer model systems covering a broad range of different properties: (i) polyethylene glycol (PEG) 20,000 is a hydrophilic and immediate release polymer, (ii) poly lactic-co-glycolic acid (PLGA) is a biodegradable sustained-release polymer, providing a drug release over several months, and (iii) ethylene-vinyl acetate (EVA) as non-biodegradable polymer with an extended-release behavior of up to 2 years. Various peptides/proteins (i.e., lysozyme, bovine serum albumin (BSA), and human insulin) were used as drug models. VCM (combination of vacuum and temperature) was used to prepare cylindrical discs composed of different protein-polymer mixtures in view of a potential as a predictive screening tool for protein solid-state stabilization in the selected polymers. The processability via VCM varied according to the used polymer: the process temperature for PEG 20,000 samples was 65 °C, whereas the samples based on PLGA 50:50 and EVA (40% VA) were produced at 70 °C. In order to study the temperature-dependent stress, the process times were selected as 6 or 12 min to obtain homogenous discs. In view of protein analysis, a combination of analytical methods, i.e., SEC (protein fragment and aggregation analysis), DSC (unfolding temperature of the protein), and FT-IR spectroscopy (conformational stability) is reported and information provided on the protein stability of the formulated and screened proteins by VCM. The applied procedure provides a good basis for the evaluation of VCM as a predictive screening tool for the preselection of appropriate polymeric matrices prior to extrusion and evaluation of protein integrity after thermal stress. On the one hand, if the process of VCM negatively affected the protein stability in protein-polymer mixtures, this combination would disqualify itself for further processing by HME. On the other hand, stable protein-polymer mixtures with sufficient protein stability after VCM could then be introduced to a combination of thermal and shear stress by HME and further investigated with regard to their process-related protein stability.

## 2. Materials and Methods

### 2.1. Materials

Human insulin was kindly donated by Sanofi Deutschland GmbH (Frankfurt am Main, Germany). Lysozyme from chicken egg-white, lyophilized powder (Cat. No. L6876) was obtained from AppliChem (AppliChem GmbH, Darmstadt, Germany). Bovine serum albumin (BSA), sodium chloride, disodium hydrogen phosphate dihydrate, sodium dihydrogen phosphate dihydrate, acetonitrile, and dichloromethane were purchased from Merck (Merck KGaA, Darmstadt, Germany). PLGA (Resomer^®^ RG502 H) was purchased from Evonik (Evonik Nutrition & Care GmbH, Darmstadt, Germany). Polyethylene glycol (PEG) 20,000 was obtained from Carl Roth (Karlsruhe, Germany). Poly(ethylene/vinyl acetate) (EVA; 60:40 (wt)) granules were purchased from Polysciences (Polysciences Inc., Warrington, PA, USA). All chemicals were of analytical grade or equivalent purity.

### 2.2. Preparation of Physical Mixtures and Sample Discs by VCM

PEG 20,000 flakes were milled utilizing a high-shear mixer (Krups Mixette type 210, Krups, Frankfurt am Main, Germany). EVA granules were milled using a cryogenic mill (6775 Freezer/Mill^®^ Cryogenic Grinder, SPEX SamplePrep LLC, Metuchen, NJ, USA). The EVA granules were loaded into the sample holder and placed in the grinding chamber, which maintains cryogenic temperatures due to continuous immersion in liquid nitrogen. EVA granules were pre-cooled for 10 min and then milled through 3 grinding cycles (10 cycles per second) at 2 min each with 2 min intercool time. For the preparation of protein-loaded discs, a physical mixture composed of polymer and 20% protein powder was manually blended with a spatula. The physical mixtures of polymer and protein powder were then vacuum compression molded. VCM was conducted using a VCM tool (MeltPrep GmbH, Graz, Austria) with a 5 mm diameter disc geometry. Approx. 15 mg of each blend was loaded into the VCM device and heated under vacuum [17] for 6 or 12 min at a temperature of 65 °C for PEG 20,000, or 70 °C for PLGA, and EVA (Table 1).

### 2.3. Sample Preparation for SEC Analysis (Protein Extraction Procedures)

Samples (i.e., physical mixture and protein-loaded discs) containing PEG 20,000 as polymer were dissolved in the mobile phase (50 mM pH 7.0 phosphate buffer, 400 mM sodium chloride) for SEC analysis (refer to Section 2.4.) corresponding to a final protein concentration of 4 mg/mL. For PLGA-containing samples, the physical mixture or protein-loaded disc was dissolved in acetonitrile. As described in the literature, proteins can be precipitated in acetonitrile without degradation of the protein structure [19]. Three cycles of protein precipitation and removal of acetonitrile by pipetting off the supernatant and evaporation of residual acetonitrile were applied to separate proteins (insoluble) from the polymer matrix (soluble). Then, 4 mg of extracted, dried proteins were pre-dissolved in 500 µL Milli-Q^®^ water (i.e., lysozyme and BSA) and diluted with 500 µL mobile phase to a target concentration of 4 mg/mL protein. Extracted human insulin was an exception, as the human insulin was firstly pre-dissolved in 0.1 M HCl and then further dissolved with the mobile phase, resulting in a final peptide concentration of 4 mg/mL. Proteins embedded in EVA were extracted by the use of dichloromethane, as described by Langer et al. [20]. The physical mixture or protein-loaded EVA discs were dissolved in dichloromethane, resulting in a precipitation of the proteins. Dichloromethane was then covered by a layer of Milli-Q^®^ water or 0.1 M hydrochloride acid in order to dissolve the precipitated proteins (lysozyme and BSA, or human insulin, respectively) by solvent extraction method. The extracted protein was collected by pipetting 500 µL of the supernatant and diluted with 500 µL of the mobile phase (corresponding to a final protein concentration of 4 mg/mL). All samples were prepared as triplicates and directly filtered into HPLC vials by syringeless filtration (Mini-UniPrep, 0.20 µm PTFE, Whatman, Cytiva, Marlborough, MA, USA) and then analyzed using the SEC method.

### 2.4. Size-Exclusion Chromatography (SEC)

Size-exclusion chromatography was performed with a Superdex 75 Increase 10/300 GL column, 10 × 300 mm (Cytiva, Marlborough, MA, USA), where the mobile phase was 50 mM pH 7.0 phosphate buffer containing 400 mM sodium chloride (isocratic) at a flow rate of 0.8 mL/min (LC-20AT, Shimadzu, Kyoto, Japan) and a column temperature of 30 °C (CTO-10AC VP, Shimadzu, Kyoto, Japan). Samples were prepared as triplicates by extracting and dissolving the samples (i.e., pure protein (reference sample 1), the physical mixture (reference sample 2), or the protein-loaded disc (VCM)) according to Section 2.3. Samples vials were cooled at 5 °C in the autosampler (SIL-A10, Shimadzu, Kyoto, Japan). Then, 10 µL of each sample was injected and potential aggregate and/or fragment formation after VCM was analyzed in comparison with a chromatogram of the freshly prepared reference solutions. The UV detection (SPD-40 UV Detector, Shimadzu, Kyoto, Japan) wavelength was 214 nm.

### 2.5. Differential Scanning Calorimetry (DSC)

DSC studies of protein powder, physical mixture, and protein-loaded discs were performed with a DSC 2 (Mettler Toledo, Gießen, Germany) equipped with an autosampler, nitrogen cooling, and nitrogen as purge gas (30 mL/min). At least three samples of ∼15 mg were accurately weighed in 40 μL aluminum crucibles with a pierced lid. DSC scans were recorded from 25 °C to 230 °C using a heating rate of 10 K/min STAR^e^ software (Mettler Toledo, Gießen, Germany) was employed for acquiring thermograms.

### 2.6. Fourier-Transform Infrared Spectroscopy (FT-IR)

FT-IR spectra were generated with a Spectrum Two^TM^ FT-IR spectrophotometer equipped with a UATR accessory (PerkinElmer, Inc., Waltham, MA, USA). A tight pressure clamp with a flat tip ensured a good contact between the sample and the reflection diamond crystal. Each sample (i.e., protein powder, physical mixture, protein-loaded discs) was measured as triplicate. The spectra were recorded against an air background between 4000 and 400 cm^−1^ with an average of four scans and a resolution of 4 cm^−1^. Data were collected in the absorption mode. First and second derivative analysis of the amide-I region (1600–1700 cm^−1^) were performed with GraphPad Prism v. 8.0.2 (GraphPad Software, La Jolla, CA, USA).

### 2.7. Statistical Analysis

Statistical analysis and testing for statistical significance were carried out using GraphPad PRISM Software (San Diego, La Jolla, CA, USA). An unpaired *t*-test (two-sample assuming equal variances) was used for the evaluation of statistical significance.

## 3. Results and Discussion

### 3.1. Unfolding Temperature of Proteins by DSC

**Figure 1:**
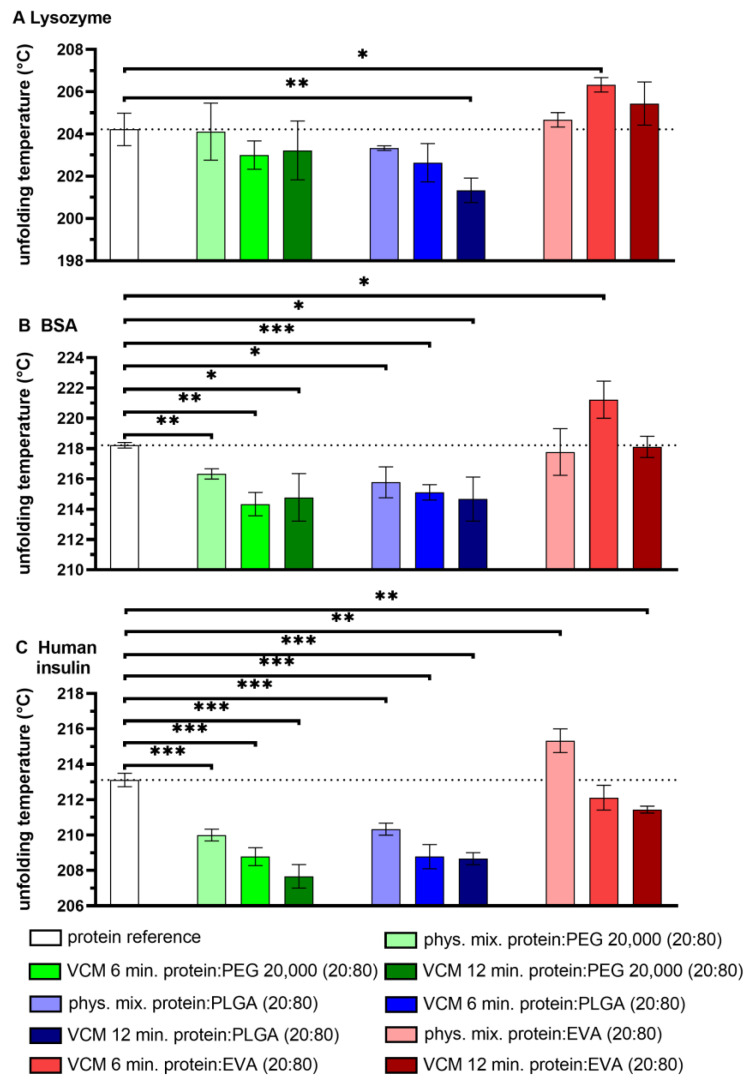
Unfolding temperature of protein powders (white), physical mixtures, and 20% protein-loaded (**A**: lysozyme; **B**: BSA; **C**: human insulin) discs with the polymers: PEG 20,000 (green) at 65 °C, PLGA (blue) at 70 °C, or EVA (red) at 70 °C prepared by VCM in 6 or 12 min; the dotted line represents the unfolding temperature of the respective unprocessed protein (reference sample); error bars represent the standard deviation of three measurements; statistical significance is depicted by asterisks (*): * *p* < 0.05, ** *p* < 0.01, *** *p* < 0.001.

The native state of a protein is its orderly folded and assembled form and determines its functionality. However, this intact structure can be altered by heat, shear stress, chemicals, or denaturants. The most common stress that can cause a loss of protein structure or functionality is an exposure to heat during processing (i.e., thermally induced protein denaturation and/or unfolding). In the case of temperature-dependent unfolding, the protein structure is lost due to elevated temperatures or a longer exposition at a high temperature [15]. We used DSC for analyzing the protein unfolding temperature in solid-state after VCM and compared it to unprocessed samples. DSC presented unfolding temperatures of native lysozyme, BSA, and human insulin as 204.7 °C, 218.2 °C, and 213.1 °C, respectively (Figure 1), which served as reference values [10]. The DSC thermograms of protein powder, pure polymers, physical mixtures composed of 20% protein powder and 80% polymer, and 20% protein-loaded discs are shown in the Supplementary Materials (Figures S1–S3). In general, the occurrence or preservation of a peak in the DSC thermogram can indicate the conservation of protein conformation after VCM, and an increasing unfolding temperature of the proteins after processing even reflects an increase in conformational protein stability. The unfolding temperatures of the physical mixtures composed of 20% protein powder and 80% PEG 20,000 or 80% PLGA were comparable with or lower than the unfolding temperature of the native proteins. However, the unfolding temperature of the proteins in the presence of EVA were not reduced or even higher compared to the native protein powder. The residence time of the molten protein-polymer mixture in the VCM tool is even higher compared to HME (e.g., mean residence time: 1 to 3 min), but 6 min was the minimum residence time for complete melting during VCM processing. The longer residence times present the worst-case scenario for when VCM should be used as a predictive screening tool for the evaluation of protein integrity for potential HME processing. VCM for 6 min highlights an optimum processing time since the unfolding temperatures of the proteins were not negatively affected compared to the unprocessed protein-polymer powder mixtures. The embedding of 20% lysozyme or BSA in 80% EVA showed a significantly increased unfolding temperature of the protein compared to the native protein powder as well as to the unprocessed physical mixture. The results indicate a potential protein stabilizing effect of EVA as polymeric matrix. Moreover, in the case of human insulin, the unfolding temperature was least negatively affected by embedding in EVA. The two-fold processing time of 12 min was not favorable since the unfolding temperatures were reduced compared to the 6 min processing time.

### 3.2. Secondary Structure Analysis and Conformational Protein Stability by FT-IR Spectroscopy

**Figure 2:**
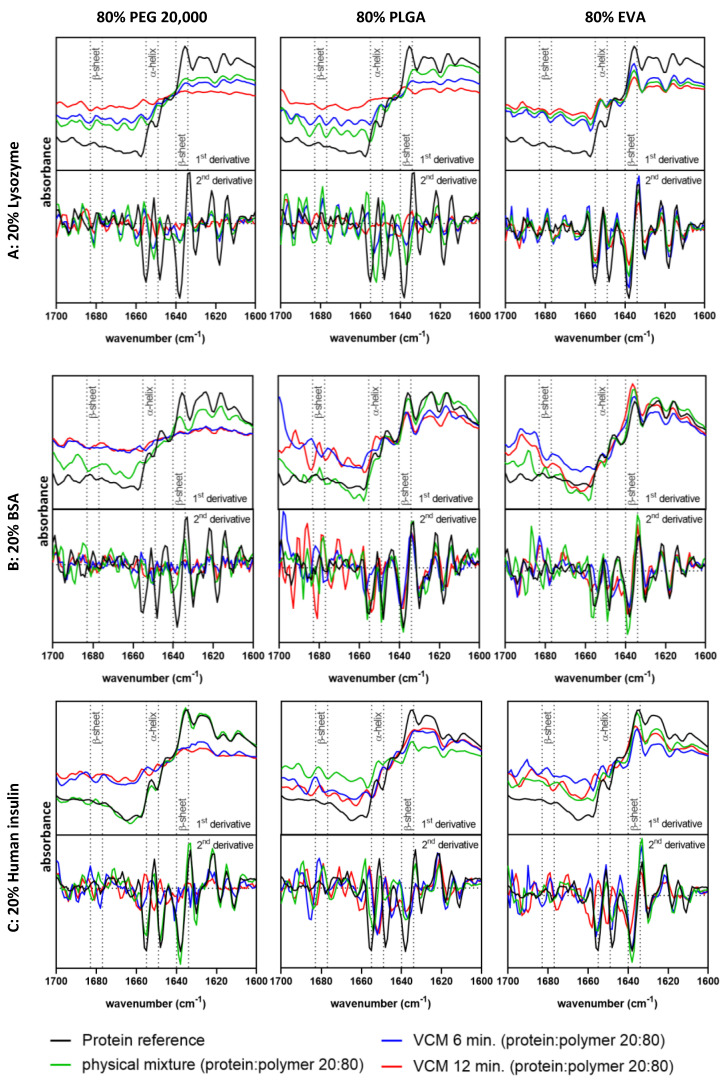
FT-IR spectra (first and second derivative) in the amide-I region (1600–1700 cm^−1^) of the respective protein powder (black); physical mixture composed of 20% protein powder and 80% polymer (green); and 20% protein-loaded (A: lysozyme, B: BSA, C: human insulin) discs with the polymers: PEG 20,000 at 65 °C, PLGA at 70 °C, or EVA at 70 °C prepared by VCM in 6 min (blue) or 12 min (red); dotted gray lines highlight secondary structure elements such as α-helices and β-sheets.

FT-IR spectroscopy was applied for comparison of the molecular protein conformation and secondary structure elements of protein powder (i.e., lysozyme, BSA, and human insulin) before and after VCM processing. The secondary structure information of the native protein powders served as a reference (Figure 2). The trend of a potential protein stabilizing effect of EVA as polymer was confirmed by FT-IR analysis. One benefit of the use of 5 mm discs was the extremely simplified analytical investigation, as the produced specimens showed a high reproducibility, were free of air inclusions, and could be directly used after production in their final form and without further sample preparation for FT-IR and DSC analysis. The first and second derivatives of spectra of VCM discs containing 20% lysozyme, BSA, or human insulin and 80% EVA were comparable to the powdered proteins used to prepare the discs. Since the spectra were overlaying, there was no indication of protein denaturation or aggregation as a consequence of the exposure to an elevated temperature of 70 °C for 6 or 12 min and vacuum during VCM processing. An exception was the formulation composed of 20% human insulin and 80% EVA processed for 12 min. Here, the spectrum was shifted compared to the reference spectra, indicating that alpha-helical structures might be disrupted due to the longer processing time at 70 °C. The use of PLGA as a polymer matrix showed no inferior effect on protein stability and was comparable to the performance of PEG 20,000. In the case of human insulin-loaded PLGA and PEG 20,000 discs, the process of VCM led to slight changes in secondary structure elements independent of the applied processing times. As discussed in a previous work, the conformational stability of lysozyme was not negatively affected by HME processing [10]. The FT-IR spectra of lysozyme-loaded PEG-extrudates produced by ram extrusion or twin-screw extrusion (TSE), as described by Dauer et al. [10], were comparable to the native lysozyme powder used to prepare the physical mixture and thus the extrudates. In contrast, the FT-IR spectra of BSA- and human insulin-loaded PEG extrudates produced by TSE showed clear shifts, when screws with a kneading element were used. The kneading element results in higher shear stress and longer residence times, to which the protein particles during processing are exposed. In particular, BSA and human insulin showed a higher susceptibility to thermal and shear stress compared to lysozyme [10]. The results of this study highlight the suitability of VCM as a screening tool for protein stability prior to HME as it follows the same trend regarding DSC and FT-IR analysis.

### 3.3. Process-Induced Formation of Protein Aggregates or Fragments by SEC

**Figure 3:**
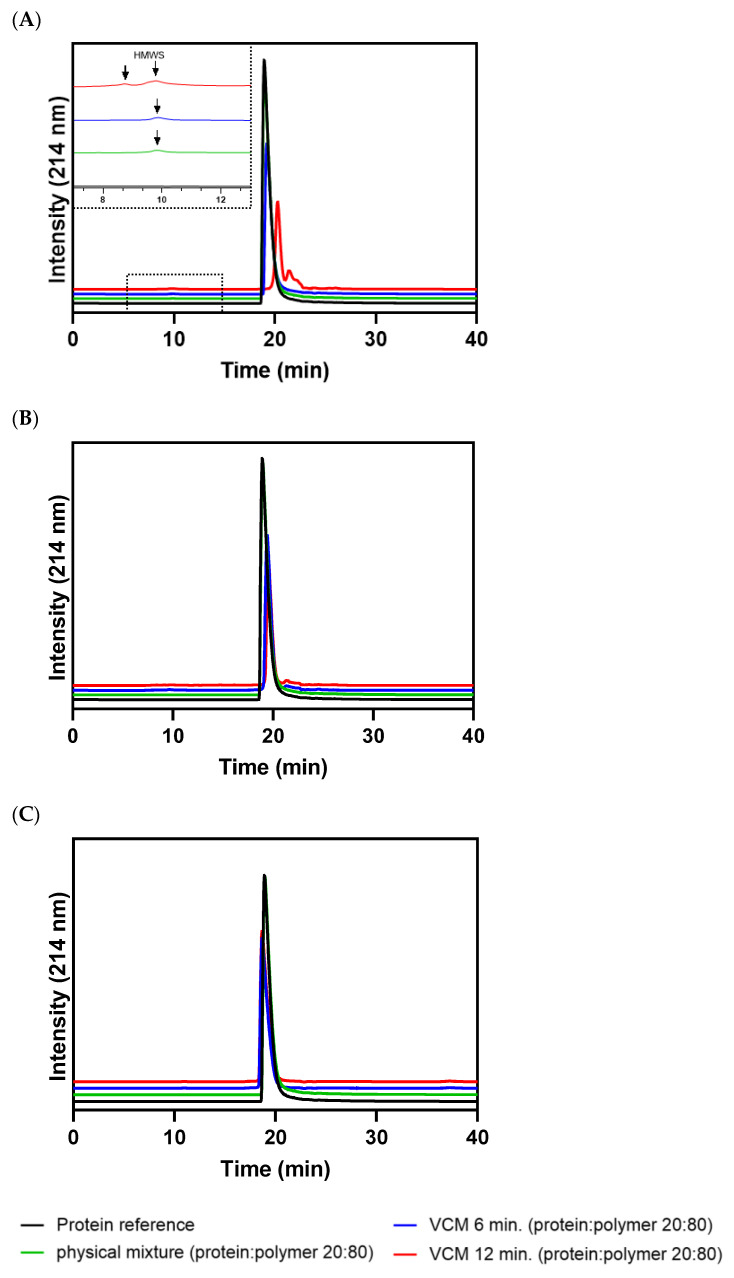
Chromatograms of SEC analysis; human insulin powder (initial: black), physical mixture composed of 20% human insulin and 80% polymer (green); and 20% human insulin-loaded discs with the polymers: (**A**): PEG 20,000 at 65 °C; (**B**): PLGA at 70 °C; (**C**): EVA at 70 °C prepared by VCM in 6 min (blue) or 12 min (red); insert: magnified area of HMWS (arrows).

Although proteins in solid-state formulations exhibit an enhanced stability, the processed proteins can undergo denaturation, degradation, or aggregation without any detectable change in their secondary structure. Consequently, structural changes of proteins cannot be distinguished from native protein species using methods such as FT-IR spectroscopy [21]. Therefore, thermal-induced protein denaturation or aggregation was investigated by SEC. The potential formation aggregation of lysozyme, BSA, and human insulin after VCM for 6 or 12 min was monitored and high-molecular weight species (HMWS)/protein aggregates or fragments were separated from native protein species. VCM processing was not the trigger for the formation of protein fragments or aggregates in the case of lysozyme- and BSA-loaded discs (Supplementary Materials: Figures S4 and S5, respectively). Lysozyme was only present as a monomer after dissolving the discs composed of 20% lysozyme and 80% PEG 20,000 in eluent, or after extraction of lysozyme from PLGA- or EVA-based discs dissolved in eluent (refer to Supplementary Materials, Figure S4). The used unprocessed and native BSA was a mixture of monomers and dimers [10]. Alteration in the ratio of monomer to dimer, or the occurrence of trimers or oligomers indicates process-related effects on BSA structure. However, the monomer-to-dimer ratio of BSA was not negatively affected by VCM (refer to Supplementary Materials, Figure S5). Native human insulin appeared as a species at a retention time of 20 min (Figure 3) and is in good agreement with previously published data [10]. Human insulin-loaded PEG discs prepared by VCM showed further peaks in front of the peak of native human insulin, indicating the formation of HMWS in the presence of PEG 20,000. With increasing process time from 6 to 12 min, the amount of HMWS was also increased. Additionally, the monomeric peak of human insulin was shifted and divided into several, non-baseline-separated small peaks. However, the formation of the HMWS was not process-related and independent of the VCM process. This is corroborated by the fact that HMWS of human insulin occurred also in the unprocessed, physical mixture (20% human insulin, 80% PEG 20,000). Therefore, PEG 20,000 is not an appropriate polymer carrier, since interactions of human insulin molecules with hydrophilic PEG chains induced the alteration of the local insulin structure [22]. This hypothesis is also confirmed by the results of human insulin-loaded extrudates prepared by ram extrusion or TSE, as described previously [10]. Human insulin-loaded PLGA discs showed a series of small peaks after the monomeric peak. Whether this observation is a polymer-, temperature-, or vacuum-related effect has to be further evaluated by an extrusion experiment. The inference is that it is a vacuum-related effect of VCM processing, since the peak areas of human insulin from PLGA discs prepared in 6 or 12 min were of the same magnitude. Human insulin extracted from EVA discs prepared in 6 or 12 min showed only a single peak at a retention time of 20 min, confirming the potential of EVA for the production of solid protein-loaded long-term release dosage forms with sufficient protein stability.

## 4. Conclusions

In this study, VCM proved to be an appropriate screening tool for the investigation of potential protein solid-state stabilization for melt-based formulations at a micro-scale as well as for the evaluation of suitable polymers and excipients for further planned HME processing. The protein-loaded discs were directly used after production in their final form for FT-IR and DSC analysis and without a further and complex sample preparation. The systematic analysis of the protein-loaded discs prepared by VCM provided important insights into the solid-state stabilizing mechanisms of protein candidates since an understanding of molecular interactions within the polymeric matrix can support the identification of most promising polymer carriers. DSC analysis proved to be a useful method for a first and rapid screening of appropriate polymers for the embedding of proteins. One benefit of using VCM as an early screening tool is to identify potential interactions between protein candidates and test polymers provoked by thermal stress during processing. Presenting short sample preparation times and flexibility regarding the manufacturing of different specimens, VCM can be used as predictive and early screening tool for extruded formulations, especially on a micro-scale level. Furthermore, VCM exploits small quantities of material and offers a “loss-less” method compared to HME, thus preventing the waste of expensive API and material in early formulation development and screening. EVA presented high potential as a polymeric matrix for solid-state stabilization of proteins (e.g., lysozyme, BSA, and human insulin) or the production of protein-loaded implants with extended-release behavior. When comparing the used polymers, EVA demonstrated better results among all selected polymers. Identified, stable protein-polymer mixtures with sufficient protein stability after VCM would then, in a next step, be introduced to a combination of thermal and shear stress by HME processing and further investigated with regard to their process-related protein stability.

## Figures and Tables

**Table 1:** Overview of the compositions and prepared protein-loaded discs by VCM.

Formulation	Components (%)		Preparation Method	Process Parameters
	Protein	Polymer		
LYS reference	100% Lysozyme			
LYS PEG PM	20% Lysozyme	80% PEG 20,000	Phys. mix.	
LYS PEG VCM 6	20% Lysozyme	80% PEG 20,000	VCM	6 min at 65 °C
LYS PEG VCM 12	20% Lysozyme	80% PEG 20,000	VCM	12 min at 65 °C
LYS PLGA PM	20% Lysozyme	80% PLGA	Phys. mix.	
LYS PLGA VCM 6	20% Lysozyme	80% PLGA	VCM	6 min at 70 °C
LYS PLGA VCM 12	20% Lysozyme	80% PLGA	VCM	12 min at 70 °C
LYS EVA PM	20% Lysozyme	80% EVA	Phys. mix.	
LYS EVA VCM 6	20% Lysozyme	80% EVA	VCM	6 min at 70 °C
LYS EVA VCM 12	20% Lysozyme	80% EVA	VCM	12 min at 70 °C
BSA reference	100% BSA			
BSA PEG PM	20% BSA	80% PEG 20,000	Phys. mix.	
BSA PEG VCM 6	20% BSA	80% PEG 20,000	VCM	6 min at 65 °C
BSA PEG VCM 12	20% BSA	80% PEG 20,000	VCM	12 min at 65 °C
BSA PLGA PM	20% BSA	80% PLGA	Phys. mix.	
BSA PLGA VCM 6	20% BSA	80% PLGA	VCM	6 min at 70 °C
BSA PLGA VCM 12	20% BSA	80% PLGA	VCM	12 min at 70 °C
BSA EVA PM	20% BSA	80% EVA	Phys. mix.	
BSA EVA VCM 6	20% BSA	80% EVA	VCM	6 min at 70 °C
BSA EVA VCM 12	20% BSA	80% EVA	VCM	12 min at 70 °C
IHU reference	100% Human Insulin			
IHU PEG PM	20% Human Insulin	80% PEG 20,000	Phys. mix.	
IHU PEG VCM 6	20% Human Insulin	80% PEG 20,000	VCM	6 min at 65 °C
IHU PEG VCM 12	20% Human Insulin	80% PEG 20,000	VCM	12 min at 65 °C
IHU PLGA PM	20% Human Insulin	80% PLGA	Phys. mix.	
IHU PLGA VCM 6	20% Human Insulin	80% PLGA	VCM	6 min at 70 °C
IHU PLGA VCM 12	20% Human Insulin	80% PLGA	VCM	12 min at 70 °C
IHU EVA PM	20% Human Insulin	80% EVA	Phys. mix.	
IHU EVA VCM 6	20% Human Insulin	80% EVA	VCM	6 min at 70 °C
IHU EVA VCM 12	20% Human Insulin	80% EVA	VCM	12 min at 70 °C

## Data Availability

No new data were created or analyzed in this study.

## Supplementary Materials

The following are available online at https://www.mdpi.com/article/10.3390/pharmaceutics15030723/s1: Figure S1. DSC thermograms (25 to 230 °C) of lysozyme powder, polymers (A: PEG 20,000; B: PLGA; and C: EVA), physical mixture composed of 20% lysozyme powder and 80% polymer, and 20% lysozyme-loaded disc produced by VCM in 6 min or 12 min. Figure S2. DSC thermograms (25 to 230 °C) of BSA powder, polymers (A: PEG 20,000; B: PLGA; and C: EVA), physical mixture composed of 20% BSA powder and 80% polymer, and 20% BSA-loaded disc produced by VCM in 6 min or 12 min. Figure S3. DSC thermograms (25 to 230 °C) of human insulin powder, polymers (A: PEG 20,000; B: PLGA; and C: EVA), physical mixture composed of 20% human insulin powder and 80% polymer, and 20% human insulin-loaded disc produced by VCM in 6 min or 12 min. Figure S4. Chromatograms of SEC analysis; lysozyme powder, physical mixture composed of 20% lysozyme and 80% polymer; and 20% lysozyme-loaded discs with the polymers: A: PEG 20,000 at 65 °C; B: PLGA at 70 °C; C: EVA at 70 °C prepared by VCM in 6 min or 12 min. Figure S5. Chromatograms of SEC analysis; BSA powder, physical mixture composed of 20% BSA and 80% polymer; and 20% BSA-loaded discs with the polymers: A: PEG 20,000 at 65 °C; B: PLGA at 70 °C; C: EVA at 70 °C prepared by VCM in 6 min or 12 min.
