# Supplementary material for: Micro-Scale Vacuum Compression Molding as a Predictive Screening Tool of Protein Integrity for Potential Hot-Melt Extrusion Processes
Source: Pharmaceutics. 2023 Feb 22;15(3):723. doi: 10.3390/pharmaceutics15030723 (PMC10054806; doi:10.3390/pharmaceutics15030723)
Supplement: Supplementary file 1 [file pharmaceutics-15-00723-s001.zip › pharmaceutics-2200720-supplementary.pdf]

pharmaceutics

MDPI

Supplementary Materials

Micro-Scale Vacuum Compression Molding as a Predictive Screening Tool of Protein Integrity for Potential Hot-Melt Extrusion Processes

Katharina Dauer and Karl G. Wagner

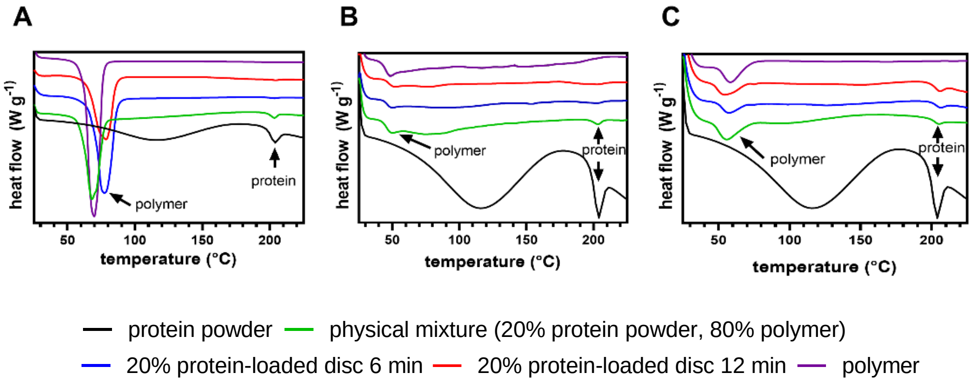

Figure S1. DSC thermograms (25 to 230°C) of lysozyme powder (black), polymers (purple) (**A**: PEG 20,000, **B**: PLGA, and **C**: EVA), physical mixture composed of 20% lysozyme powder and 80% polymer (green), and 20% lysozyme-loaded disc produced by VCM in 6 min (blue) or 12 min (red).

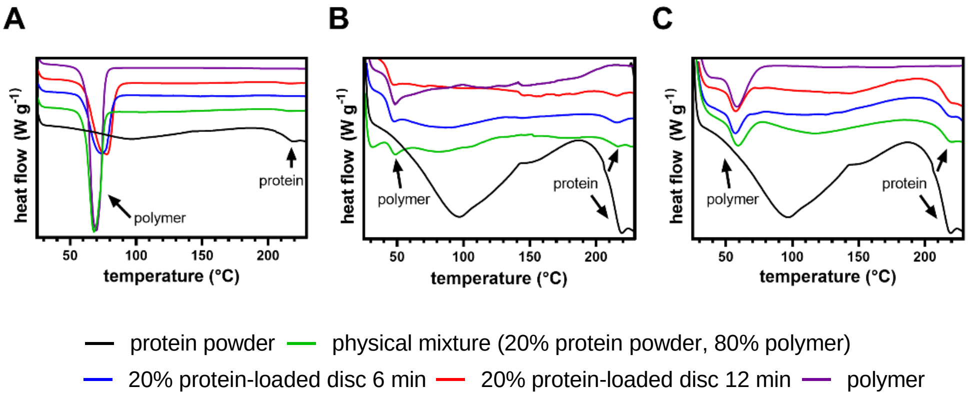

Figure S2. DSC thermograms (25 to 230°C) of BSA powder (black), polymers (purple) (**A**: PEG 20,000, **B**: PLGA, and **C**: EVA), physical mixture composed of 20% BSA powder and 80% polymer (green), and 20% BSA-loaded disc produced by VCM in 6 min (blue) or 12 min (red).

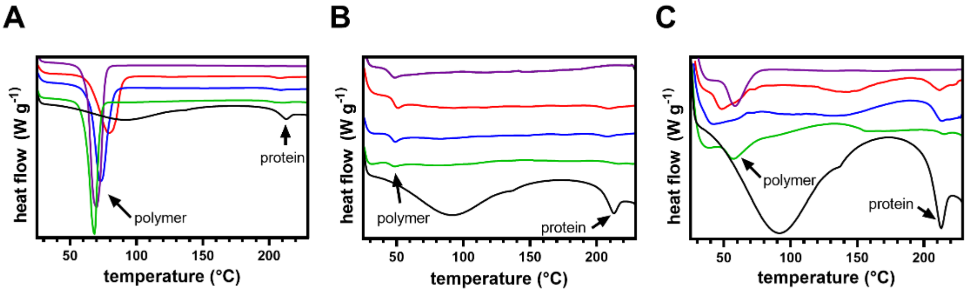

— protein powder — physical mixture (20% protein powder, 80% polymer)
— 20% protein-loaded disc 6 min — 20% protein-loaded disc 12 min — polymer

Figure S3. DSC thermograms (25 to 230°C) of human insulin powder (black), polymers (purple) (**A**: PEG 20,000, **B**: PLGA, and **C**: EVA), physical mixture composed of 20% human insulin powder and 80% polymer (green), and 20% human insulin-loaded disc produced by VCM in 6 min (blue) or 12 min (red).

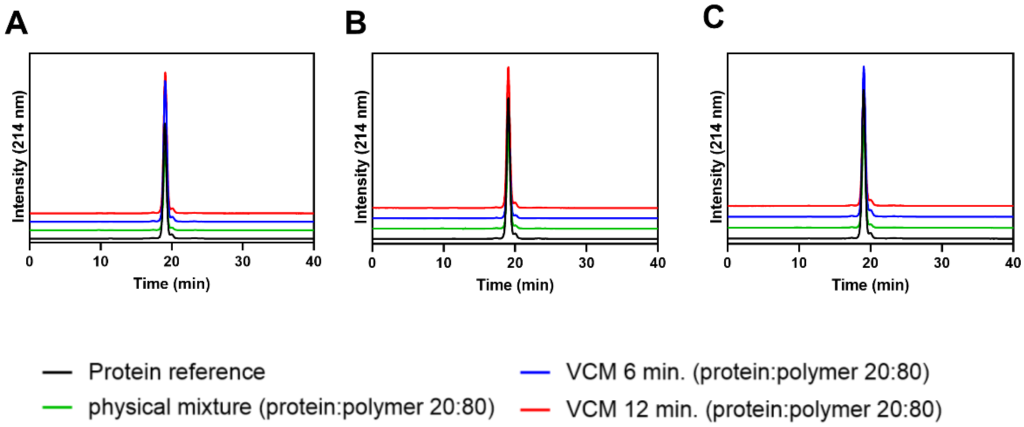

Figure S4. Chromatograms of SEC analysis; lysozyme powder (initial: black), physical mixture composed of 20% lysozyme and 80% polymer (green); and 20% lysozyme-loaded discs with the polymers: **A:** PEG 20,000 at 65°C; **B:** PLGA at 70°C; **C**: EVA at 70°C prepared by VCM in 6 min (blue) or 12 min (red).

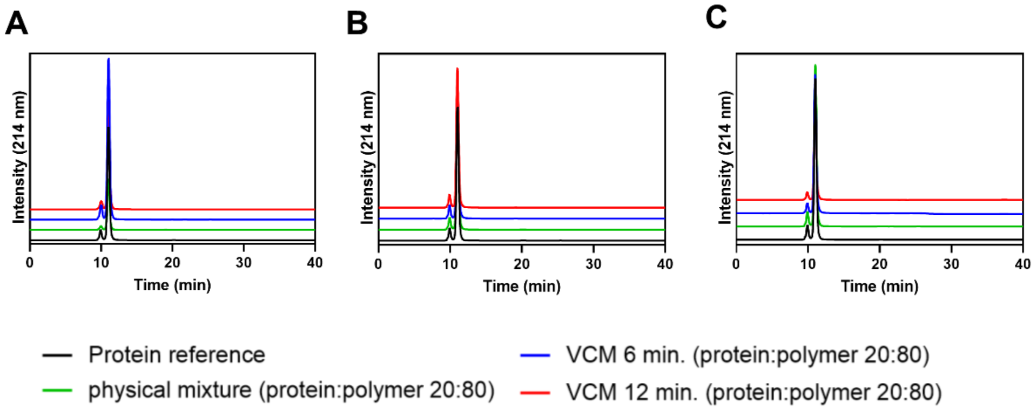

Figure S5. Chromatograms of SEC analysis; BSA powder (initial: black), physical mixture composed of 20% BSA and 80% polymer (green); and 20% BSA-loaded discs with the polymers: **A:** PEG 20,000 at 65°C; **B:** PLGA at 70°C; **C**: EVA at 70°C prepared by VCM in 6 min (blue) or 12 min (red).
